# Correction of Multiple Canine Impactions by Mixed Straightwire and Cantilever Mechanics: A Case Report

**DOI:** 10.1155/2014/925019

**Published:** 2014-07-22

**Authors:** Sergio Paduano, Iacopo Cioffi, Giorgio Iodice, Vincenzo d'Antò, Francesco Riccitiello, Gioacchino Pellegrino, Rosa Valletta

**Affiliations:** ^1^Department of Health, University “Magna Graecia” of Catanzaro, Viale Europa, Loc. Germaneto, 88100 Catanzaro, Italy; ^2^Department of Neurosciences and Oral Sciences, University of Naples Federico II, Via Pansini 5, 80131 Naples, Italy

## Abstract

*Background.* This case report describes the orthodontic treatment of a woman, aged 17 years, with a permanent dentition, brachyfacial typology, Angle Class I, with full impaction of two canines (13,33), and a severe ectopy of the maxillary left canine. Her main compliant was the position of the ectopic teeth. *Methods.* Straightwire fixed appliances, together with cantilever mechanics, were used to correct the impaired occlusion and to obtain an ideal torque control. *Results and Conclusion.* The treatment objectives were achieved in 26 months of treatment. The impactions were fully corrected with an optimal torque. The cantilever mechanics succeeded in obtaining tooth repositioning in a short lapse of time. After treatment, the dental alignment was stable.

## 1. Introduction

The impaction of canines is a frequent report in clinical practice. Previous studies report frequencies ranging between 1% and 3% for the maxillary canines [1] and about 2% for the mandibular cuspids [2]. The palatal impaction of maxillary cuspids is about four times greater than labial impactions [3]. Furthermore, canine impaction occurs more frequently unilaterally than bilaterally [4, 5].

The aetiology of canine impaction is generally related to the upper dental arch length deficiency or to the developmental position of the tooth that could be more cranial than the norm. Mechanical factors have also been proposed to be related to the impaction of a canine. For instance, the premature loss of deciduous canines or their prolonged retention could be associated with mechanical impingement of permanent canine. This can also be the consequence of displacement of lateral incisors or of the presence of impacted teeth or odontomas [4, 5].

Untreated impacted canines may determine arch length discrepancies, loss of vitality of adjacent teeth, follicular cysts, canine ankylosis, infections, and pain [6]. As a consequence of this, orthodontic treatment is strongly recommended.

Cantilever mechanics have been proposed for orthodontic recovery of impacted teeth. A two-tooth system stressed by a couple of forces can be used to obtain an extrusive force to the canine and an intrusive force to the molar. One-couple orthodontic appliances can increase the predictability of tooth movement and reduce the need of appliance reactivation and the occurrence of possible intra-arch unwanted side effects [7, 8].

We present the case of a 17-year-old girl with a permanent dentition (Figure 1), brachyfacial typology, Angle Class I, with full impaction of two canines (13,33), severe ectopic eruption of the maxillary left canine, and posterior crossbite. Her main compliant was the position of the ectopic teeth. Straightwire fixed appliances, together with cantilever mechanics, were used to correct the impaired occlusion and to obtain an ideal torque control.

## 2. Case Report

The patient presented this objective problem list:

- Class 1 skeletal and dental malocclusion in the permanent dentition,
- full crossbite extended from the maxillary left deciduous canine to the first permanent molar,
- ectopic eruption of the maxillary left canine,
- palatal impaction of the maxillary right canine,
- impaction of the mandibular left canine due to the presence of an odontomas,
- discrepancy of the midlines of upper and lower dental arches.

The cephalometric evaluation highlighted a brachyfacial typology with a sagittal skeletal relationship of Class I (Figure 2). The panoramic radiograph showed the germs of wisdom teeth of both dental arches (Figure 2). A palatal impaction of the maxillary right canine, the ectopic eruption of the maxillary left canine, and the impaction of the mandibular left canine due to a small odontoma were visible in the CT scan (Figure 3). The patient did not present signs or symptoms of temporomandibular disorders according to Research Diagnostic Criteria for Temporomandibular Disorders (RDC/TMD) [9].

The treatment plan included the correction of crossbite, aligning and levelling by straightwire fixed appliance, management of the space for impacted and ectopic teeth by coil springs, surgical exposure, recovery of impacted and ectopic teeth by cantilevers, torque control of recovered teeth, and finishing.

Initially, a TPA proclination spring was scheduled [10] for obtaining a buccal movement of the maxillary incisors. However, the patient reported a slight discomfort. As a consequence of this, it was removed and a conventional multibracket treatment was planned.

A .036′′ stainless steel transpalatal arch was modelled to obtain unilateral expansion of the maxillary left side. Alignment of both dental arches was achieved by using multibracket appliance (Roth prescription, slot size .022′′ × .028′′) with heat activated Ni-Ti archwires (round .014′′ and round .016′′). The space for impacted and ectopic teeth was obtained by using superelastic coil springs on round .018′′ A.J Wilcock Australian wire (regular+, G&H Orthodontics, Franklin, IN, USA).

A flap for the surgical exposure of the impacted maxillary left canine was obtained by an intrasulcular incision extended from the first right maxillary incisor to the second upper premolar of the same side. Once exposed, the palatal surface of the tooth was etched for 30 seconds and rinsed with water. Transbond XT (3M Unitek Monrovia, USA) adhesive primer was used for its strength [11, 12] following the instruction of the manufacturer. The lingual sheath was anchored to a .012′′ stainless steel ligature and bonded onto the distal palatal surface of the tooth, in order to prevent unwanted rotations during buccal movement. Eyelets were created within the ligature for a proper cantilever insertion. A flap was then used to remove the odontoma and to expose the mandibular left canine. A lingual sheath was bonded using the same procedure described above.

The cantilever was modelled using .019′′ × .025′′ TMA wire (Ormco, Orange, CA, USA, Figure 4). TMA is an elastic titanium molybdenum alloy showing Young's modulus of about 100 GPa [13]. Initially, a first order bend—offset (a) was modelled close to the molar to avoid interferences with premolar brackets. A second order bend (b) was then modelled to activate extrusion. Finally, a first order bend (c) directed palatally was used to anchor the canine. A hook was bended at the end of the cantilever to allow a proper tooth engagement.

The extrusion of the maxillary right canine was obtained by inserting a third order bend (read as a buccal root torque of the molar) at the insertion of the maxillary molar (Figures 5(a) and 5(c)). This activation leads to a caudal displacement of the hook at the boundary of the cantilever. The force delivered was about 150 grams, as measured by means of a dynamometer. This cantilever was also activated to obtain a buccal movement of the tooth by activating the first order bend close to the molar.

For the maxillary left canine the cantilever was activated in extrusion by a second order bend (Figures 5(b) and 5(c)), which determined a caudal displacement of the hook. Also for this case, the force delivered was about 150 grams. To avoid buccal root torque of the maxillary right molar and distal tipping of the left maxillary molar, the transpalatal arch was kept in place. Also this reduced unwanted side effects, such as mesiopalatal rotation of the right first maxillary molar, related to the buccal activation of the cantilever in the right side. To obtain additional anchorage, a second transpalatal arch was placed on the maxillary second molars.

A straight cantilever was modelled for the mandibular left canine, because of the good position of the tooth. A second order bend was modelled close to the molar for obtaining extrusion (Figure 5). To further obtain an adequate torque of the canines, during treatment, a bracket with −17° torque (left second mandibular premolar) was used to obtain a palatal root torque of the maxillary left canine. For the maxillary right canine, a similar bracket was bonded but turned 180°, to obtain a buccal root torque.

The progressive orthodontic recovery of both maxillary and mandibular canines is documented in Figures 6 and 7.

The archwire treatment considered superelastic nickel-titanium (Ni-Ti) archwires. These materials undergo phase transition driven by temperature and stress. The main feature of this alloy is its capability to release an almost constant level of stress during the orthodontic treatment [14]. The archwire treatment sequence included the following:

- .014′′ HA Ni-Ti (heat-activated) upper and lower alignment archwires (3M Unitek, Monrovia, CA, USA);
- .016′′ HA Ni-Ti upper and lower alignment archwires (3M Unitek, Monrovia, CA, USA);
- .018′′ AJ Wilcock Australian archwire regular+ with coil springs for space opening;
- .018′′ × .025′′ HA Ni-TI upper and lower archwires (3M Unitek, Monrovia, CA,USA);
- .019′′ × .025′′ SS upper and lower archwires (3M Unitek, Monrovia, CA,USA);
- .018′′ × .025′′ multibraded wires with vertical elastics (1/8′′, 4 Oz) for improving intercuspation.

A translation utility arch (TRUA) was also used for upper incisors retraction preserving an ideal torque [15]. During repositioning of the canines, the patient experienced slight discomfort.

## 3. Results and Discussion

A patient with multiple impaction of canines and unilateral posterior crossbite extended from the left maxillary first molar to the deciduous canine was successfully treated by a combination of cantilever mechanics and straightwire appliance. The patient affected with crossbite did not present temporomandibular disorders [16]. The extraoral and intraoral photographs of the patient at the end of treatment are reported in Figure 8. The total treatment duration was 26 months. Patient compliance was high throughout the treatment and good oral hygiene was maintained. The cantilever mechanics allowed a correct repositioning of the ectopic and impacted canines. However, since the force was applied on a single point of the canines, it did not allow for a proper correction of their torque [7]. Hence, it was decided to use extratorque brackets for a proper torque control. Molar and canine Class I relationships were obtained as well as a proper overjet and overbite. The facial profile was improved due to the correction of the incisor torque, which resulted in the decreased nasolabial angle. The repositioning of the maxillary canines provided better lip support as shown in Figures 8 and 9. Panoramic and lateral radiographs with cephalometric tracing before debonding at the end of treatment and tracing superimpositions are reported in Figures 9 and 10.

For this orthodontic treatment we decided to use conventional brackets because it has been suggested that passive self-ligating brackets may be less effective for obtaining an adequate torque control [17–19]. Also we used heat activated Ni-Ti archwires to reduce patient discomfort [20]. Ferric-sulphate gel for bleeding control in surgical exposure of impacted canines was also used, in order to reduce postoperative pain [21].

A lower fixed retainer was bonded from the mandibular right canine to left canine to maintain lower incisor alignment. Post-treatment photographs at two-year follow-up are presented in Figure 11. The results achieved were maintained during the retention period by means of the fixed lingual retainer that has not been removed yet.

## Figures and Tables

**Figure 1:**
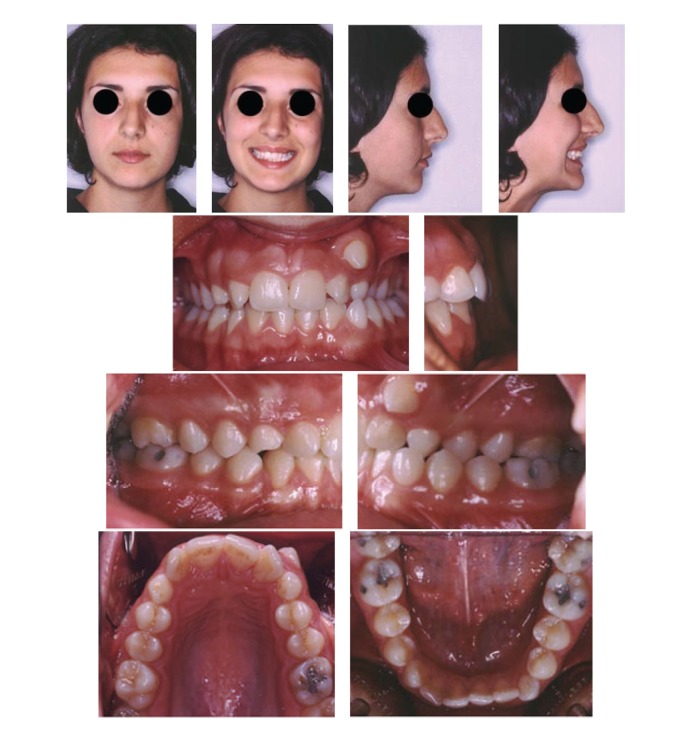
Extraoral and intraoral photographs before treatment.

**Figure 2:**
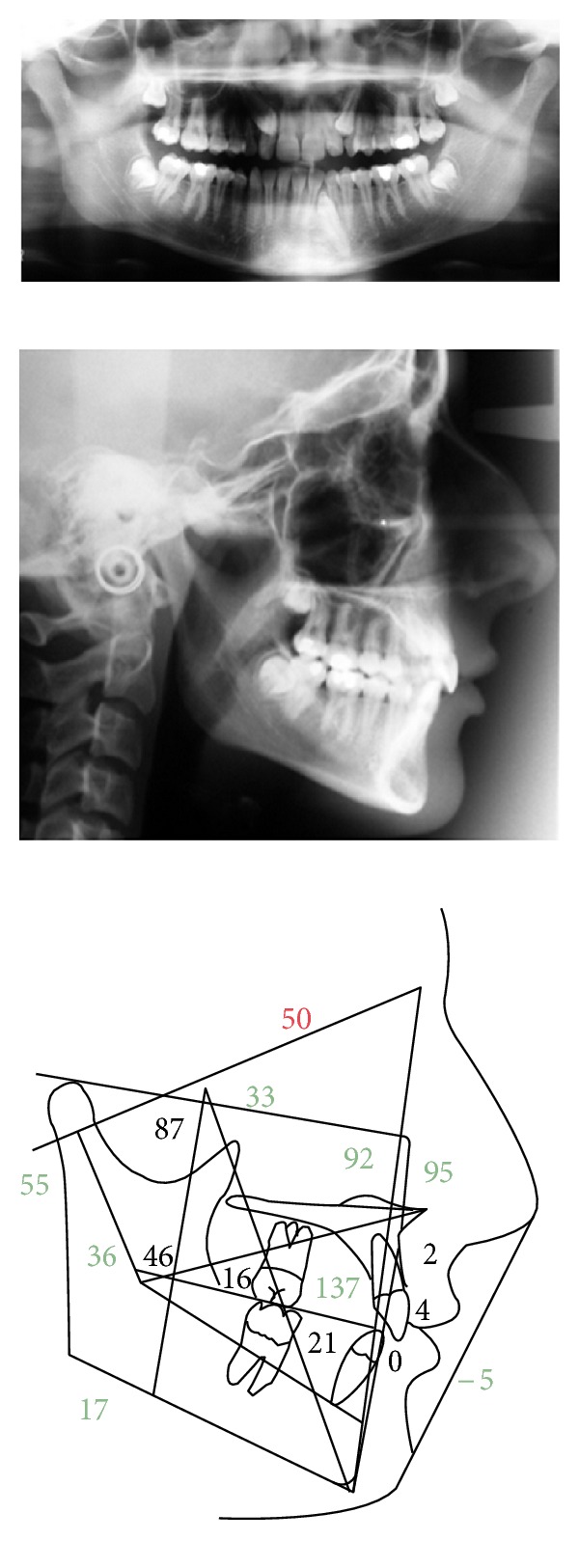
Cephalometric values and panoramic radiograph at the start of treatment.

**Figure 3:**
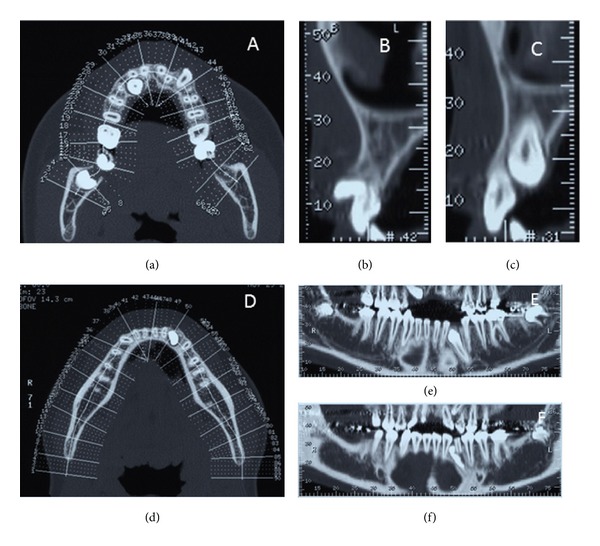
CT scan showing the axial (a) and the sagittal ((b), (c)) view of the maxillary impacted canine and the axial and frontal view of the mandibular canine ((d), (e), (f)).

**Figure 4:**
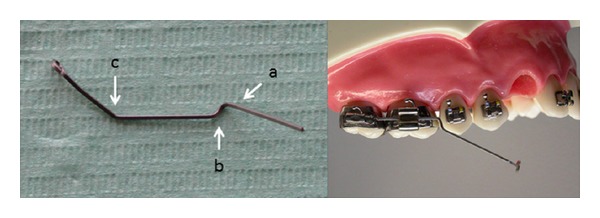
A cantilever modelled using .019′′ × .025′′ TMA. Initially, a first order—offset (a) and a second order (b) bends are modelled close to the maxillary molar to avoid interferences with premolar brackets and to activate the cantilever. Then, a first order bend (c) directed palatally is used to anchor the canine.

**Figure 5:**
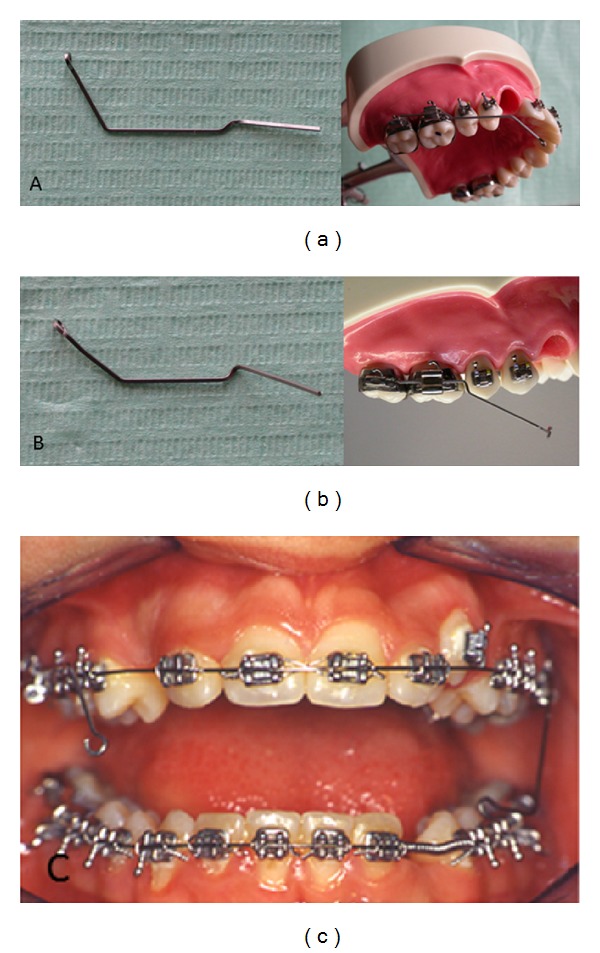
Cantilever activated for obtaining extrusion by a third order bend (a) and a second order bend (b). Cantilevers in position (c).

**Figure 6:**
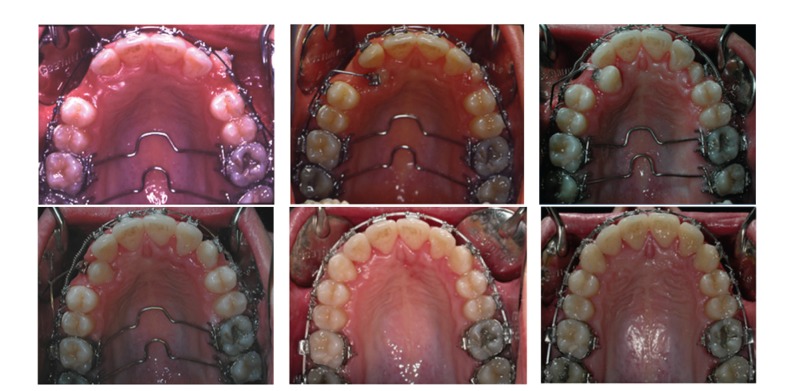
From left to right, progressive recovery of the maxillary canines.

**Figure 7:**
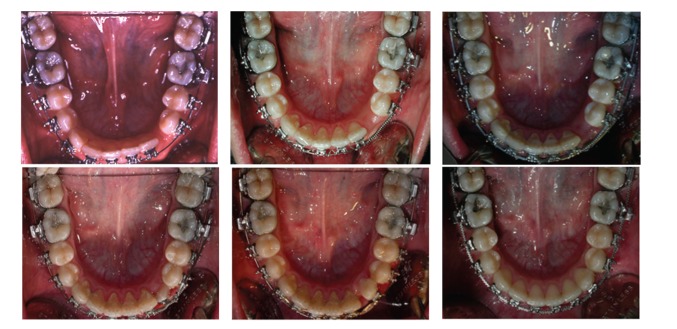
From left to right, progressive recovery of the mandibular canine.

**Figure 8:**
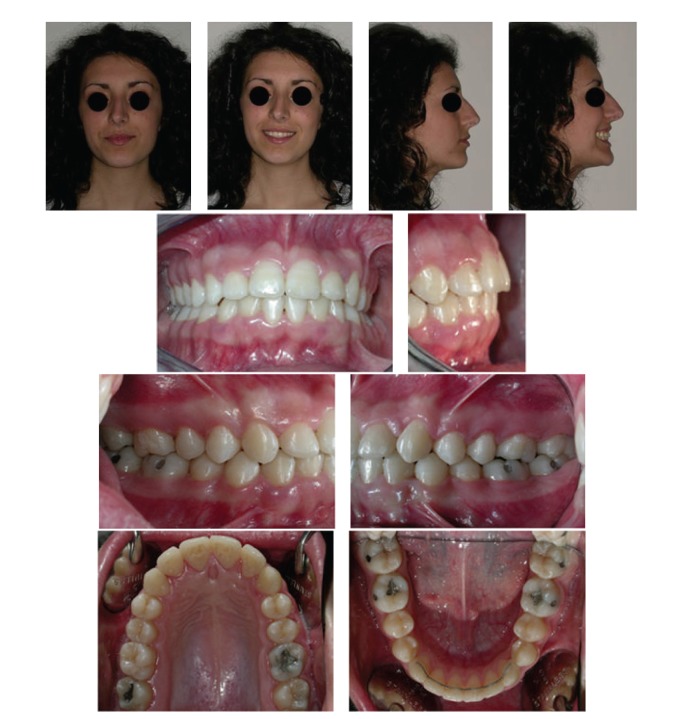
Extraoral and intraoral photographs after treatment.

**Figure 9:**
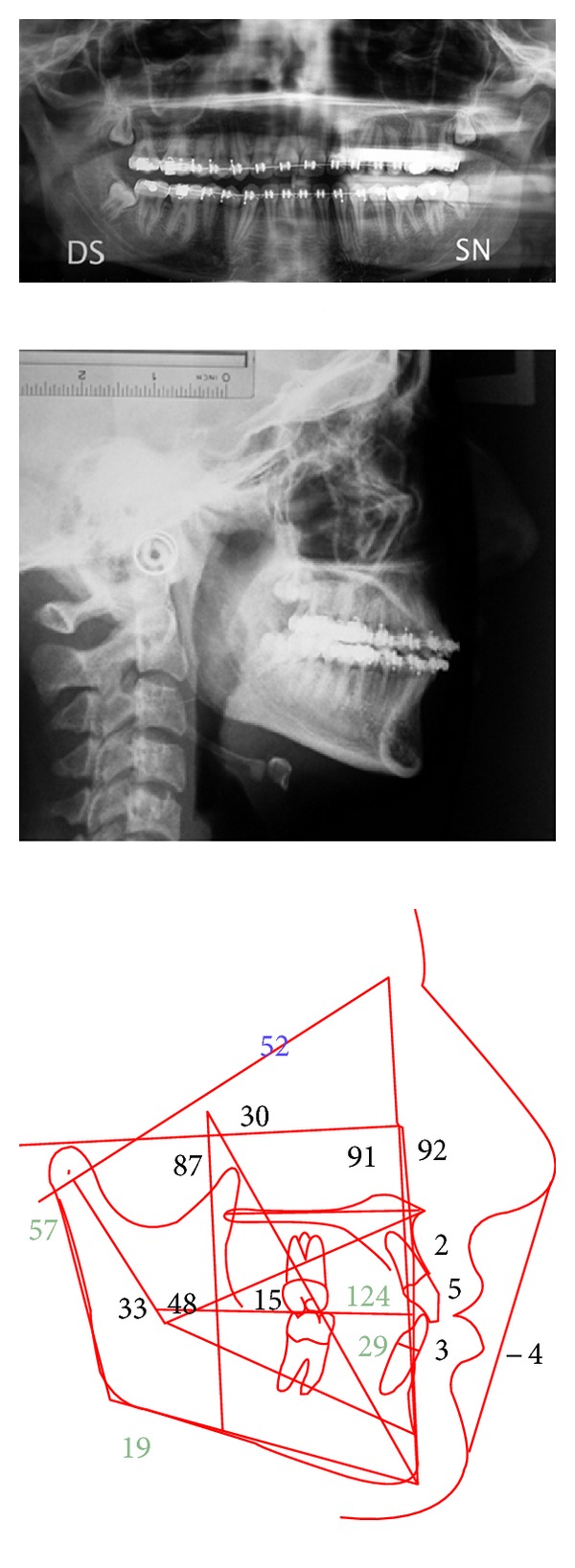
Panoramic and lateral radiographs with cephalometric tracing before debonding at the end of treatment.

**Figure 10:**
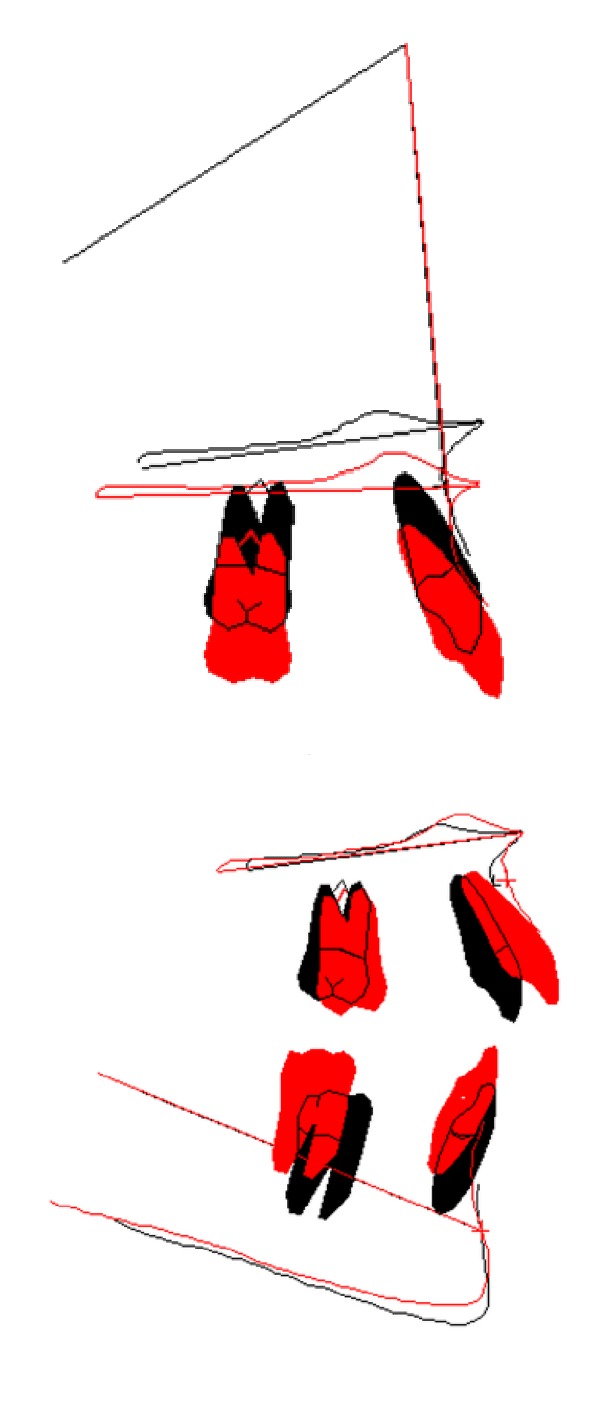
Superimpositions of the upper and lower dental arches before (black) and after (red) treatment.

**Figure 11:**
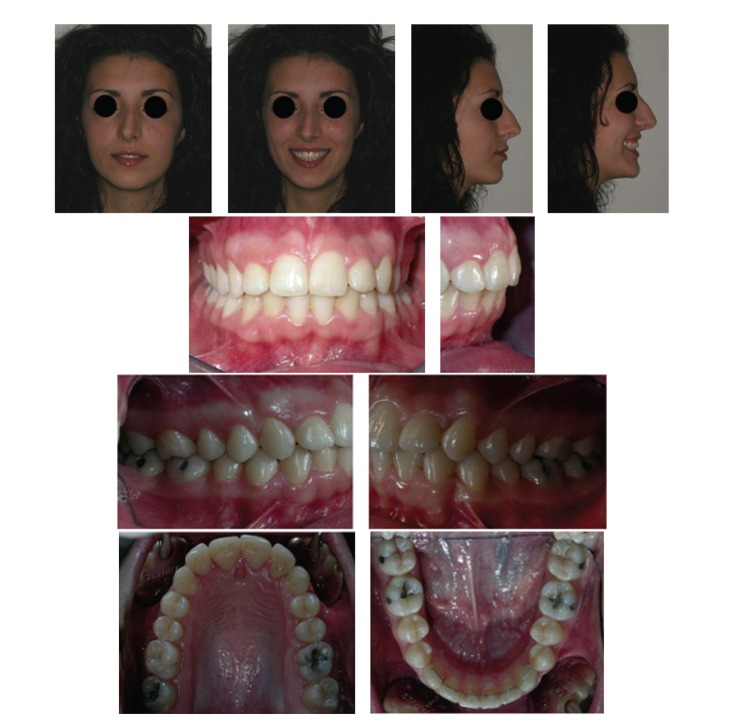
Extraoral and intraoral photographs at two-year follow-up.
